# Late-onset fabry disease presenting with unexplained renal failure, left ventricular hypertrophy, and recurrent syncope: a case report

**DOI:** 10.1186/s13023-025-03791-4

**Published:** 2025-06-03

**Authors:** Qisu Ying, Xiu Yang, Ning Zhao, Ming Wang, Yu Wu, Xuexue Shi, Jie Shen, Min Zhao, Wenjun Wang, Yingying Qian, Qi Chen, Yong Mao

**Affiliations:** 1 Department of Nephrology, Hangzhou First People’s Hospital, Hangzhou, Zhejiang China; 2 Department of Nephrology, Hangzhou First People’s Hospital Chengbei Campus, Hangzhou Geriatric Hospital, Hangzhou, Zhejiang China; 3 Department of Pharmacy, Hangzhou First People’s Hospital, Hangzhou, Zhejiang China; 4 Department of Ultrasound, Hangzhou First People’s Hospital, Hangzhou, Zhejiang China; 5 Department of Gastroenterology, Hangzhou First People’s Hospital Chengbei Campus, Hangzhou Geriatric Hospital, Hangzhou, Zhejiang China

**Keywords:** Fabry disease, Renal failure, Left ventricular hypertrophy, Recurrent Syncope, Hearing loss

## Abstract

Late-onset cardiac manifestations of Fabry disease are frequently associated with high rates of missed diagnoses and misdiagnoses. We present a case of a 71-year-old male with late-onset Fabry disease whose diagnosis was delayed due to the absence of typical symptoms. The patient has a history of nephrotic syndrome and is currently suffering from end-stage renal disease (ESRD), undergoing maintenance hemodialysis. He was previously diagnosed with diffuse left ventricular hypertrophy and heart block. Upon admission, cardiac examination revealed reduced longitudinal strain of the left ventricle. Fabry disease was suspected due to recurrent heart failure, persistent slight elevation in troponin I (TNI) levels, recurrent syncope, and hearing loss. Subsequent measurement of α-galactosidase A activity and genetic testing confirmed the diagnosis. This case highlights the importance of considering Fabry disease in patients with renal failure, recurrent heart failure, persistent slight elevation in TNI levels, and bilateral interventricular septum syndrome.

## Introduction

Fabry disease (FD), also known as Anderson-Fabry disease or systemic angiokeratoma, is a rare X-linked lysosomal storage disorder caused by mutations in the GLA gene, which encodes the alpha-galactosidase A enzyme [1, 2]. The disease is characterized by the progressive accumulation of glycosphingolipids, including globotriaosylceramide (GL-3), in the lysosomes of the cells of multiple tissues and organs. FD can have a multi-systemic involvement leading to cardiac, cutaneous, ocular, neurological, renal, and/or gastrointestinal (GI) manifestations with the cardiac variant being the most common variant of late-onset FD. As GL-3 levels rise, cardiomyocytes develop hypertrophy, inflammatory infiltrates, and interstitial fibrosis, and lesions can affect cardiomyocytes, the myocardial interstitium, the conduction system, valves, and blood vessels [3, 4]. Over time, these alterations lead to complications such as arrhythmias, left ventricular hypertrophy (LVH), valvular abnormalities, myocardial fibrosis, and heart failure. However, distinguishing LVH in FD from other cardiac conditions, such as hypertension, valvular disease, hypertrophic cardiomyopathy (HCM), or infiltrative diseases like amyloidosis, can be clinically challenging. Due to the nonspecific and atypical symptoms associated with the cardiac variant of late-onset FD, early diagnosis is often delayed, which significantly impacts timely treatment and overall prognosis.

This article presents a case of a patient with heart and kidney failure secondary to late-onset FD, whose diagnosis was delayed due to the absence of typical clinical manifestations.

## Case presentation

A 71-year-old male patient was diagnosed with stage 5 chronic kidney disease and type 2 diabetic nephropathy at a local hospital two years ago. After undergoing left forearm arteriovenous fistula (AVF) formation surgery, the patient initiated maintenance hemodialysis treatment. During this period, he experienced recurrent episodes of chest tightness, intermittent syncope, and mild chest pain.

The patient’s renal history began in 2018 with isolated proteinuria and mild hematuria, typical of nephrotic syndrome. From 2018 to 2022, urine tests showed 3+/4 + proteinuria (no red blood cells), 24-hour protein 4260–8050 mg, albumin 15.8–32.5 g/L, and urine volume 1200–3500 ml/day. Serum creatinine rose from 88.6 µmol/L (2018) to 267.8 µmol/L (2021) and 440 µmol/L (2022), requiring hemodialysis. Lab data are in Table 1.

**Table 1:** The patient’s laboratory test results

	2018	2019	2020	2021	2022
Urine Protein	(+++)	(++++)	(++++)	ND	(+++)
Urine Red Blood Cells	(-)	(-)	ND	ND	(-)
Urine Specific Gravity	1.023	1.011	ND	ND	1.022
Urine Glucose	2+	(+-)	ND	ND	3+
24-hour Urine Volume (mL)	2000	1500–3500	1200–1600	1200	ND
24-hour Urine Protein Quantitation (mg/24 h)	ND	4852.6–8050	4260–8050	5738.4	ND
Blood Albumin (g/L)	26.8–29	25.8–28.8	15.8	30.4	32.5
Blood Creatinine (µmol/L)	88.6-109.1	108–141	155.9	267.8	440

Sixteen years ago, the patient was diagnosed with coronary atherosclerotic heart disease and had his first stent placement surgery (details unknown). Since then, he underwent 9 stent placements and 3 coronary drug-eluting balloon treatments. The patient was also diagnosed with left ventricular hypertrophy and conduction blocks (left anterior fascicular block, complete right bundle branch block), with ECG showing poor R-wave progression in the anterior lateral wall and biphasic or inverted T-waves in high lateral leads. Cardiovascular exam history is in Table 2.

**Table 2:** The history of cardiovascular examination results

Year	Age	Symptoms	Evaluation	Main Diagnosis	Management
2019	66	Chest tightness	**TTE**: Diffuse thickening of left ventricular wall, hypertrophic cardiomyopathy (non obstructive type), left Ventricular diastolic dysfunction	Coronary atherosclerotic heart disease, hypertrophic cardiomyopathy	Control high blood pressure, antiplatelet therapy
2021	68	Chest tightness	**ECG**: Sinus rhythm, complete right bundle branch block, left anterior branch block, mild ST segment elevation (0.1 mV horizontal ST segment elevation in V3 and V4 leads). **TTE**: Increased left atrial diameter, thickening of left ventricular wall, mild pulmonary hypertension	Coronary atherosclerotic heart disease, hypertrophic cardiomyopathy	Control high blood pressure, antiplatelet therapy
2022	69	Chest tightness	**Coronary CTA**: Severe stenosis in the proximal lumen of the left anterior descending artery stent, mild stenosis in the lumen of the right coronary artery, heart enlargement with small amount of pericardial effusion.	Coronary atherosclerotic heart disease	Control high blood pressure, antiplatelet therapy
2024.1	71	Chest tightness	**CAG**: Segmental calcification with stenosis of 70% in the proximal mid segment of the right coronary stent, stenosis of 50% in the distal segment, TIMI blood flow grade 3. Left main trunk stenosis of 30%. 30% stenosis in the proximal and middle segments of the anterior descending branch, 60–70% stenosis in the distal segment of the circumflex branch, and TIMI blood flow grade 3.	Coronary atherosclerotic heart disease	Two drug balloons were deployed on the proximal stent of the right coronary artery for dilation
2024.8	71	Chest tightness	**ECG**: Complete right bundle branch block, left anterior branch block, V2-V3 abnormal q waves, increased left ventricular comprehensive voltage, T wave changes (negative positive bidirectional or inverted in leads I, aVL, V4), clockwise transposition.	Fabry disease	Isosorbide Mononitrate and Nifedipine Controlled Release Tablets

ECG: Electrocardiogram, TTE: Transthoracic echocardiogram, CAG: coronary angiogram, CTA: CT angiography, TIMI: thrombolysis in myocardial infarction

In the past two years, the patient had recurrent melena and abdominal pain, diagnosed with gastrointestinal bleeding. Gastroscopy in 2019 showed an esophageal varix, reflux esophagitis (Grade A), and erosive chronic non-atrophic gastritis. Repeat gastroscopy at our hospital revealed multiple gastric ulcers with scarring.

**Fig. 1:**
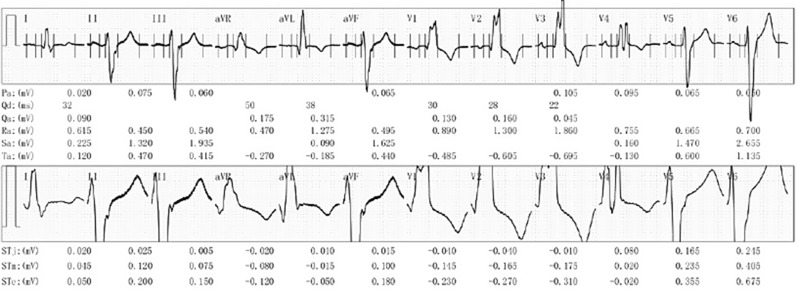
Holter monitoring showed dual-origin atrial premature beats, complete right bundle branch block and left bundle branch block

**Fig. 2:**
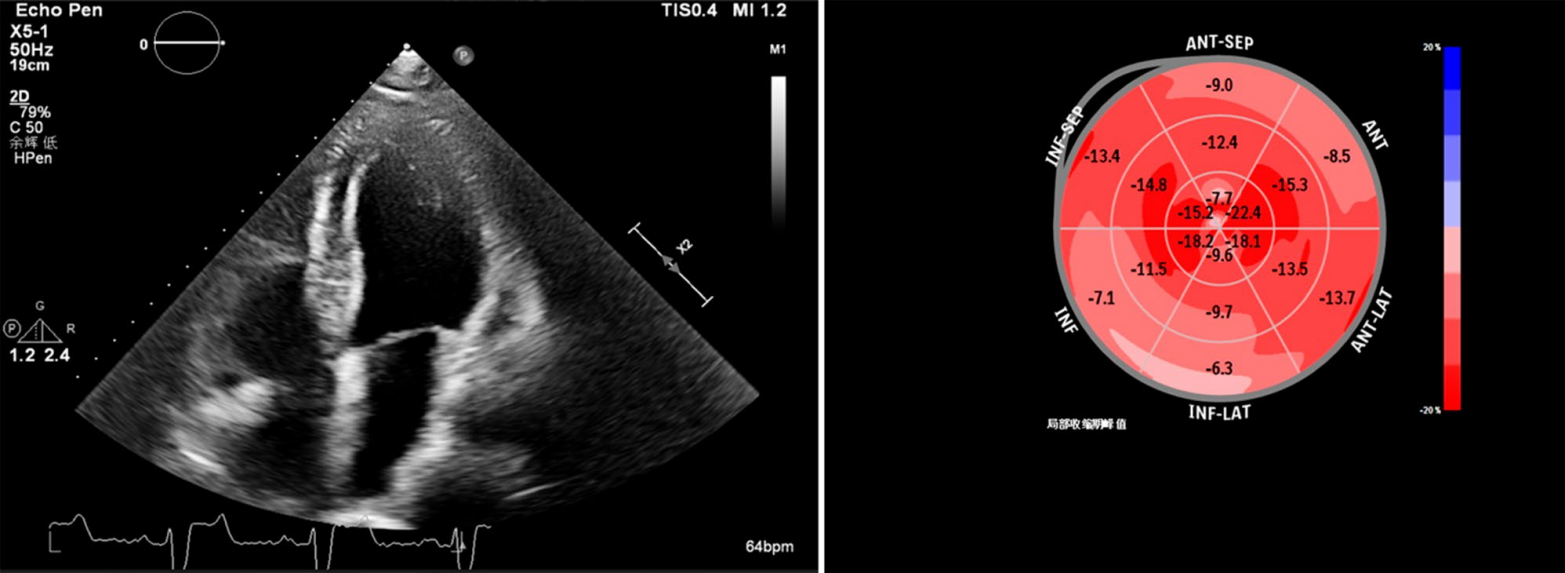
The three-dimensional echocardiography revealed a distinct bilateral sign in the ventricular septum, diffuse hypertrophy of the left ventricular wall, left ventricular dysfunction

During hospitalization in April 2024, the patient had three syncope episodes. Cranial CT was normal. The cranial MRI showed a few lacunar foci in the bilateral lateral ventricles. Holter monitoring (Fig. 1) showed sinus rhythm with low max heart rate, dual-origin atrial premature beats, and combined right/left bundle branch blocks. 3D echocardiography (Fig. 2) revealed a ventricular septal bilateral sign, diffuse left ventricular hypertrophy (1.9–2.0 cm), left ventricular dysfunction (LVEF: 0.45, E/e: 33.1), and reduced left ventricular global longitudinal strain (LV-GLS: -9%, normal: males − 17%~-25%). Cardiac MRI was unobtainable due to poor cooperation. Cardiogenic syncope was ruled out.

During the neurological examination, we observed that the knee tendon reflex was absent, the muscle strength in the lower extremities was grade 4, and electromyography suggested widespread peripheral neuropathy (both sensory and motor nerves were involved, with demyelinating damage combined with axonal damage). we tested for ganglioside antibodies and paraneoplastic syndrome antibodies, both of which were negative.

**Fig. 3:**
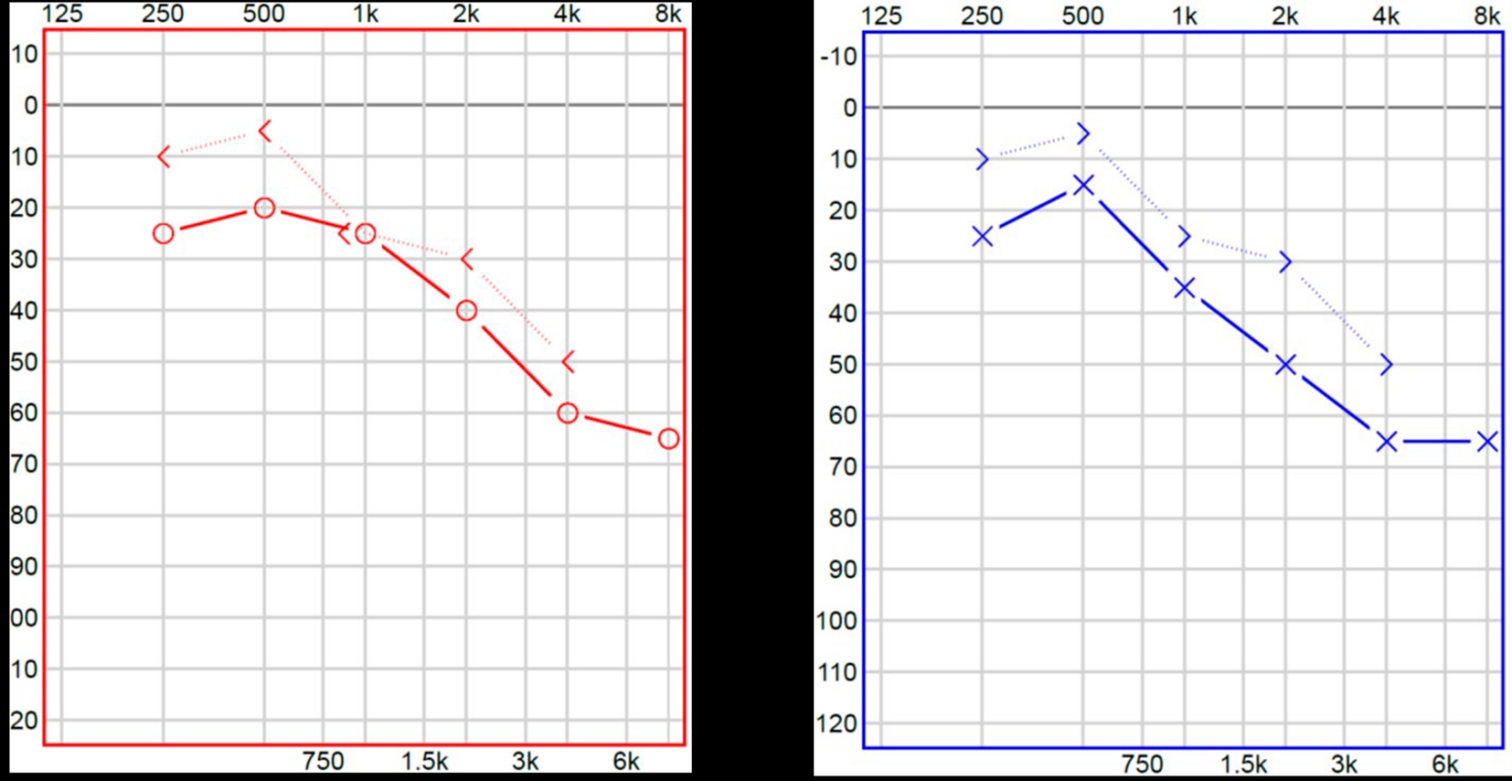
A Pure tone audiogram: pure tone average (PTA) between 0.25–8 kHz of 28 dB HL on the right side and of 33 dB HL on the left side

The patient reported a gradual decline in hearing over the past year. Pure-tone audiometry revealed sensorineural high-frequency hearing loss (see Fig. 3).

In summary, the patient presented with multi-systemic manifestations, including cardiac abnormalities (conduction block, coronary heart disease, myocardial hypertrophy), hearing impairment (high-frequency hearing loss), nephropathy (nephrotic syndrome, renal failure), gastrointestinal symptoms (recurrent bleeding, abdominal distension), and recurrent syncope. Differential diagnoses considered were amyloidosis, Fabry disease, and vasculitis. Further tests and imaging ruled out the diagnosis of amyloidosis (negative results of serum free light chain assay and abdominal fat biopsy) and vasculitis (negative results of Antineutrophil Cytoplasmic Antibodies).

Evaluation for Fabry disease included lysosomal storage disease screening, which showed significantly reduced α-galactosidase A activity (0.94 µmol/L/h; reference range: 2.20–17.65 µmol/L/h) and elevated Lyso-GL-3 (4.42 ng/ml; reference range: <1.11 ng/ml) (see Table 3). These findings strongly support a diagnosis of Fabry disease.

**Fig. 4:**
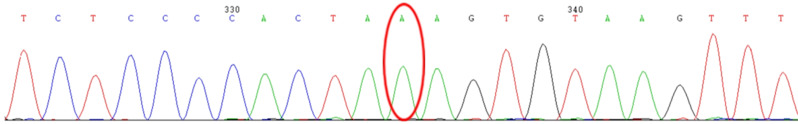
The Sanger diagram of genetic testing suggests that the subject has detected GLA: NM-000169.3: c.640-801G > A mutation, also known as IVS4 + 919G > A mutation, which is a splicing mutation. This mutation is generally detected in patients with late-onset Fabry disease

**Fig. 5:**
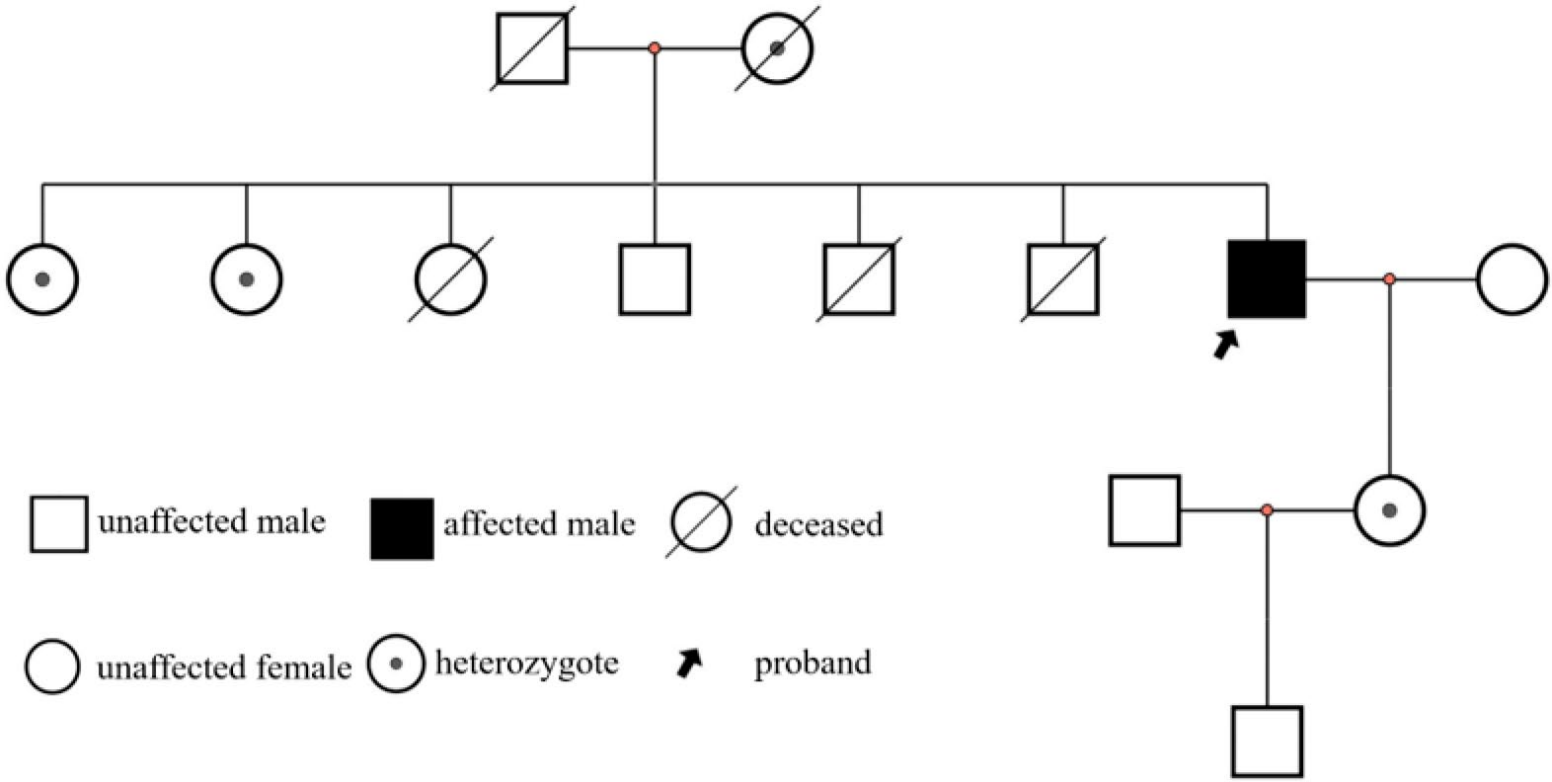
The patient’s family genealogy chart

Genetic testing for definitive diagnosis identified the splicing variant GLA: NM_000169.3:c.640-801G > A (IVS4 + 919G > A), a variant frequently associated with late-onset Fabry disease (see Table 3). The patient was ultimately diagnosed with Fabry disease based on genetic testing and clinical manifestations. The Sanger sequencing results are shown in Fig. 4, and the patient’s family pedigree chart is illustrated in Fig. 5.

**Table 3:** Examination related to Fabry disease

Examination	Results	Reference Value
α-Gal A activity	0.94 µmol/L/h	2.20-17.65 µmol/L/h
Lyso-GL-3	4.42 ng/ml	< 1.11 ng/ml
Genetic sequencing	(c.604-801G > A) heterozygous variant encoding intron 4 of the GLA gene (NM_000169.3)	Compatible with FD

Following 3 months of enzyme replacement therapy (ERT) with recombinant alpha-galactosidase A, the patient experienced significant relief of abdominal pain and bloating, with no recurrence of gastrointestinal bleeding and persistently negative fecal occult blood tests. Follow-up cardiac ultrasound revealed marked improvement in left ventricular ejection fraction (LVEF, 0.61), while three-dimensional echocardiography demonstrated increased stroke volume and a mild enhancement in left ventricular global longitudinal strain (GLS, from − 9% to -10.2%). No episodes of syncope occurred during ERT. Audiometry revealed no further decline in bilateral hearing. Repeat electromyography showed no progression of axonal and demyelinating injuries in both lower limbs. During lifelong maintenance of ERT, continuous follow-up for multisystem involvement remains necessary.

## Discussion

Fabry disease is a rare genetic disorder that affects approximately one in every 40,000 to 110,000 individuals. Lin et al. [5] screened ~ 57,000 newborn boys and found various Fabry mutations in ~ 1 in 1400, 83% of whom had the cardiac variant mutation IVS4 + 919G>A, for an incidence of ~ 1 in 1600. Hwu et al. [6] screened ~ 90,000 baby boys and found Fabry mutations in ~ 1 in 1250, 86% with IVS4 + 919G>A, an incidence of 1 in 1500.

Symptoms of Fabry disease include a burning sensation in the hands and feet, reduced sweating, nausea, abdominal pain, postprandial diarrhea, and developmental disorders [7], as well as progressive hearing loss [8]. When the kidneys are affected, symptoms include hematuria, nephrotic syndrome, and renal failure [9]. Although the disease manifests in childhood, it is often misdiagnosed or missed [7]. This may be due to the lack of characteristic clinical manifestations in patients or to clinicians’ insufficient awareness of multisystem lysosomal storage diseases. The patient in this case lacked typical clinical manifestations such as skin angiokeratomas, limb pain, and reduced sweating. His nephrotic syndrome was attributed to diabetic nephropathy, and his heart disease was considered to be caused by coronary atherosclerotic heart disease. His hearing loss, syncope, abdominal distension, and other issues were frequently overlooked by physicians, resulting in a delay in diagnosis for over a decade. Cardiac involvement in Fabry disease can manifest as hypertension, myocardial ischemia due to coronary artery involvement, valvular abnormalities, and hypertrophic cardiomyopathy. In severe cases, it may present as angina pectoris, myocardial infarction, and heart failure, which are often late-stage manifestations of the disease and the primary causes of death [4]. The clinical manifestations in this patient included hypertension, coronary heart disease, recurrent heart failure, recurrent arrhythmias, and angina pectoris. Echocardiography revealed diffuse hypertrophy of the left ventricular wall and segmental motion abnormalities.

The patient had been undergoing hemodialysis for three years when he was admitted to our hospital. Upon initially taking over the patient’s case, we meticulously reviewed his past medical history and noted that his renal function had deteriorated more rapidly than is typically seen in patients with diabetic nephropathy. This prompted us to investigate the potential link between the patient’s renal disease and his other systemic manifestations. Systemic diseases such as amyloidosis, syphilis-related nephropathy, and systemic lupus erythematosus were excluded through a comprehensive panel of investigations, including serum free light chain assay, syphilis serology, antinuclear antibody (ANA) panel testing, and abdominal fat biopsy. Hereditary kidney diseases should not be overlooked, as some Fabry disease patients may present with coronary artery lesions and heart conduction abnormalities due to cardiac involvement, and some may exhibit nephrotic syndrome [10]. However, Fabry disease typically manifests in younger individuals, and this patient was already 71 years old. We believed that the possibility of late-onset Fabry disease could not be ruled out, so We conducted tests for alpha-galactosidase A and lyso-GL-3, which revealed abnormal results. Consequently, we proceeded with GLA gene testing, and the results indicated a splicing mutation, leading to a definitive diagnosis of Fabry disease.

When Fabry disease presents without typical clinical manifestations and is accompanied by underlying conditions such as diabetes and hypertension, it is easy to attribute heart failure, myocardial hypertrophy, and renal failure to diabetes and hypertension, while overlooking the possibility of hereditary kidney diseases. Meanwhile, the patient’s renal failure was considered to be caused by nephrotic syndrome. To explain the etiology of the patient’s nephrotic syndrome, we believed that the patient might have diabetic kidney disease (DKD), but the possibility of non-diabetic kidney disease (NDKD) could not be ruled out. The patient’s renal function was normal in 2018, but a large amount of urinary protein was already present at that time, and it rapidly progressed to uremia within 2–3 years. This did not exclude the possibility of chronic kidney disease (CKD) combined with acute kidney injury (AKI), and we believed that AKI might also be related to recurrent heart failure and the repeated use of contrast agents during heart catheterization. Whether the patient’s nephrotic syndrome was related to Fabry disease could only be confirmed through renal biopsy. The typical pathological manifestations of Fabry disease include vacuolar degeneration of glomerular visceral epithelial cells, generally no immune complex deposition in immunofluorescence, and characteristic findings such as osmiophilic myeloid bodies under electron microscopy [9].

Fabry disease can cause non-specific gastrointestinal symptoms, such as malabsorption, delayed gastric emptying, reduced intestinal motility, and ischemic or neuropathic damage. It appears that the three primary mechanisms implicated in this context are the dysfunction of the autonomic nervous system that governs gut motility, the vasculopathy impacting gastrointestinal circulation, and the tissue inflammation associated with GL-3 accumulation [11, 12]. In individuals afflicted with Fabry disease, it appears that small-caliber nerve fibers, whether thinly myelinated or unmyelinated, which are implicated in thermal and pain perception, autonomic innervation, and the enteric nervous system, tend to sustain damage [13]. Patient in this case primarily presented with abdominal bloating, slowed intestinal motility, and recurrent gastrointestinal bleeding. Gastric mucosal biopsy revealed moderate chronic inflammation with focal intestinal metaplasia, and electron microscopy did not identify the characteristic ultrastructural pathological changes of Fabry disease. Therefore, we cannot establish a direct correlation between the abdominal symptoms and Fabry disease. We also need to consider whether the patient’s fainting and dizziness symptoms, as well as cerebral infarction, are related to Fabry disease. Related literature shows that Fabry disease is an important risk factor for stroke [14]. Glycosphingolipids can further deposit into the endothelial cells and microvascular smooth muscle cells of cerebral blood vessels, leading to stenosis and thrombosis of small arteries, thereby causing ischemic stroke and dizziness. The patient has a history of carotid artery stenosis (moderate stenosis at the beginning of the right external carotid artery, with a stenosis rate of 50–69%; stenosis of the left common carotid artery). Therefore, it cannot be ruled out that these symptoms may be due to cerebral vascular insufficiency caused by carotid artery stenosis.

Indeed, there are reports in the literature that Fabry disease can present with symptoms such as dizziness and syncope, sometimes leading to a diagnosis in neurology clinics. Fabry disease is an important risk factor for stroke [15]. The mechanism may involve the further deposition of glycosphingolipids into the endothelial cells and microvascular smooth muscle cells of cerebral blood vessels, leading to stenosis and thrombosis of small arteries, thereby causing ischemic stroke and dizziness. Some individuals with Fabry disease may present with demyelinating disease, neuropathy, and stroke [16], which can sometimes be confused with multiple sclerosis. However, the mechanism by which Fabry disease mimics multiple sclerosis has not been well evaluated [17]. We should have performed a lumbar puncture to examine the cerebrospinal fluid for a definitive diagnosis, but this was not carried out due to the patient’s refusal.

It is challenging to determine whether the patient’s progressive hearing loss is caused by Fabry disease, as advanced age and chronic renal failure are often associated with hearing loss. The mechanism by which Fabry disease damages hearing is currently unclear, but it is speculated that it may be due to the deposition of glycosphingolipids in the inner ear or intracranial blood vessels.

Numerous studies on Fabry disease associated with the IVS4 + 919G > A mutation have primarily focused on Taiwanese and Chinese populations, resulting in incomplete understanding of its global epidemiological characteristics and phenotypic variability. Critical future research should include: (1) conducting cross-ethnic genotype-phenotype correlation studies; and (2) establishing an international registry integrating multi-omics data to address current sample limitations. Furthermore, extended follow-up periods using standardized metrics are required to clarify the impact of enzyme replacement therapy (ERT) on multi-organ disease progression.

## Conclusions

Fabry disease has a higher prevalence among individuals with kidney diseases (such as chronic kidney disease, undergoing dialysis), heart diseases (including left ventricular hypertrophy, hypertrophic cardiomyopathy), and neurological diseases (like ischemic stroke). By screening high-risk populations, the diagnostic rate of Fabry disease can be improved, and effective treatment measures can be promptly implemented to prevent the occurrence of severe complications. Particular attention should be paid when high-risk individuals are simultaneously accompanied by non-specific symptoms of Fabry disease, such as high-frequency hearing impairment, recurrent syncope, and gastrointestinal bleeding.

## Data Availability

The data used to support the findings of this study are included within the article.

## Abbreviations

ESRD: End-stage renal disease
TNI: Troponin I
FD: Fabry disease
GLA: Alpha-galactosidase A
GL-3: Globotriaosylceramide
GI: Gastrointestinal
LVH: Left ventricular hypertrophy
HCM: Hypertrophic cardiomyopathy
AVF: Arteriovenous fistula
ECG: Electrocardiogram
TTE: Transthoracic echocardiogram
CAG: Coronary angiogram
CT: Computed Tomography
CTA: CT angiography
MRI: Magnetic Resonance Imaging
GLS: Global Longitudinal Strain
ANA: Antinuclear Antibodies
DKD: Diabetic kidney disease
NDKD: Non-diabetic kidney disease
CKD: Chronic kidney disease
AKI: Acute kidney injury
PTA: Pure-tone average
